# Atrial Fibrillation and Documented Heart Failure in Patients Hospitalized with COVID-19: A Secondary Analysis of a Single-Centre Cohort from Western Romania

**DOI:** 10.3390/jcm15166470

**Published:** 2026-08-21

**Authors:** Ana-Maria Pah, Cristiana Adina Avram, Maria Rada, Gheorghe Stoichescu-Hogea, Adina Bucur, Dan Alexandru Surducan, Abdeldayem Mahmoud, Ovidiu Calin Ilie, Claudiu Avram, Emilia Elena Clej, Petra Alexandra Lazureanu, Maria-Laura Craciun

**Affiliations:** 1 Department of Cardiology, “Victor Babeș” University of Medicine and Pharmacy of Timișoara, Eftimie Murgu Square 2, 300041 Timișoara, Romania; anamaria.pah@umft.ro (A.-M.P.); rada.maria@umft.ro (M.R.); laura.craciun@umft.ro (M.-L.C.); 2 Department of Internal Medicine I, “Victor Babeș” University of Medicine and Pharmacy of Timișoara, Eftimie Murgu Square 2, 300041 Timișoara, Romania; 3 Department of Functional Sciences, Discipline of Public Health, Center for Translational Research and Systems Medicine, “Victor Babeș” University of Medicine and Pharmacy of Timișoara, Eftimie Murgu Square 2, 300041 Timișoara, Romania; bucur.adina@umft.ro (A.B.); surducan.dan@umft.ro (D.A.S.); 4 Centre for Translational Research and Systems Medicine, Faculty of Medicine, “Victor Babeș” University of Medicine and Pharmacy of Timișoara, Eftimie Murgu Square 2, 300041 Timișoara, Romania; 5 Department of Family Medicine, “Victor Babeș” University of Medicine and Pharmacy of Timișoara, Eftimie Murgu Square 2, 300041 Timișoara, Romania; mahmoodemadsalama@gmail.com; 6 Department I, Discipline of Dental Prostheses Technology (Dental Technology), Faculty of Dental Medicine, “Victor Babeș” University of Medicine and Pharmacy of Timișoara, 300041 Timișoara, Romania; ilie.ovidiu@umft.ro; 7 Department XVI—Balneology, Medical Rehabilitation and Rheumatology, “Victor Babeș” University of Medicine and Pharmacy of Timișoara, Eftimie Murgu Square 2, 300041 Timișoara, Romania; avram.claudiu@umft.ro; 8 Transdisciplinary Research Center for Medical Rehabilitation, Balneology and Rheumatology (CTRMBR), “Victor Babeș” University of Medicine and Pharmacy of Timișoara, Eftimie Murgu Square 2, 300041 Timișoara, Romania; 9 Doctoral School, Department of General Medicine, “Victor Babeș” University of Medicine and Pharmacy of Timișoara, Eftimie Murgu Square 2, 300041 Timișoara, Romania; emilia.clej@umft.ro; 10 Faculty of Medicine, “Victor Babeș” University of Medicine and Pharmacy of Timișoara, Eftimie Murgu Square 2, 300041 Timișoara, Romania; petra.lazureanu@student.umft.ro

**Keywords:** atrial fibrillation, documented heart failure, COVID-19, cross-sectional analysis, cardiovascular–kidney–metabolic complexity, multimorbidity, Romania

## Abstract

**Background/Objectives**: Atrial fibrillation (AF) and heart failure (HF) frequently coexist, but later-pandemic data from Eastern Europe are limited. We evaluated their documented coexistence in patients hospitalized with COVID-19; the study was not designed to determine whether SARS-CoV-2 modifies the established AF–HF relationship. **Methods**: We retrospectively analysed 395 adults admitted with RT-PCR-confirmed SARS-CoV-2 infection between 1 September 2022 and 31 December 2024. AF was identified from the admission record, while HF was determined by an audited, rule-based review of cardiovascular free-text entries. A modified Poisson model with robust variance was the primary analysis and estimated adjusted prevalence ratios (aPRs) after adjustment for age, sex, body mass index, hypertension, ischaemic heart disease, pre-existing type 2 diabetes, chronic kidney disease, chronic obstructive pulmonary disease, smoking, and prior ischaemic stroke. Logistic regression and a restrictive NYHA-coded HF definition were sensitivity analyses. **Results**: AF was documented in 68 patients (17.2%), HF in 106 (26.8%), and both conditions in 26 (6.6%). HF prevalence was 38.2% among patients with AF and 24.5% among those without AF. AF was associated with documented HF in the primary model (aPR 1.51, 95% confidence interval 1.06–2.16; *p* = 0.022); age was also associated with HF (aPR 1.02 per year, 95% confidence interval 1.01–1.04; *p* = 0.010). Results were similar with the restrictive HF definition (aPR 1.54, 95% confidence interval 1.07–2.23) and logistic regression (adjusted odds ratio 1.89, 95% confidence interval 1.06–3.37). Exploratory mortality estimates were imprecise and were not used for prognostic inference. **Conclusions**: AF identified a subgroup with a higher prevalence of documented HF within this hospitalized COVID-19 cohort. The findings are best interpreted as evidence of cardiovascular and multimorbidity complexity, not as proof of a COVID-specific, temporal, or causal AF–HF effect.

## 1. Introduction

Cardiovascular involvement is a major determinant of clinical complexity in hospitalized COVID-19. Although direct myocardial infection may occur, most acute cardiac manifestations are interpreted in the context of systemic inflammation, hypoxaemia, endothelial dysfunction, autonomic disturbance, and decompensation of pre-existing cardiac disease [1,2,3].

Atrial fibrillation is the most frequently reported sustained arrhythmia in hospitalized COVID-19 [4,5]. Its reported prevalence varies with case mix and ascertainment: pooled estimates are approximately 8% overall and 11% for pre-existing AF, with both categories associated with adverse short-term outcomes [6,7,8,9].

AF and HF also have a well-established bidirectional relationship. AF can worsen ventricular function through loss of atrial contraction, rapid or irregular ventricular response, and remodelling, whereas HF promotes AF through elevated filling pressures, atrial stretch, fibrosis, and neurohormonal activation [10,11,12,13,14].

Acute infection may unmask or destabilize this shared cardiovascular substrate through hypoxaemia, inflammation, and haemodynamic stress [15,16,17,18,19,20]. However, without a non-COVID comparator, a hospitalized COVID-19 cohort cannot establish that SARS-CoV-2 modifies the AF–HF relationship. Advanced age and cardiovascular comorbidity also remain major determinants of adverse outcomes in hospitalized COVID-19 populations [21,22,23].

The specific gap addressed here is descriptive and contextual: most published COVID-19 AF data derive from early pandemic waves, and later-pandemic Eastern European cohorts remain underrepresented [7,8,9,24,25].

Related analyses from the same institution have examined sepsis-associated cardiac biomarkers [26], pulmonary embolism [27], and acute pulmonary oedema [28]; the present analysis addresses a distinct question concerning documented AF–HF coexistence.

We therefore examined the cross-sectional association between documented AF and documented HF in 395 adults hospitalized with COVID-19 at a tertiary infectious diseases centre in Western Romania. The primary objective was to estimate the adjusted prevalence ratio for coexisting documented HF among patients with versus without AF. Recorded NYHA class and in-hospital mortality were secondary descriptive outcomes. Because there was no non-COVID comparator and both diagnoses were ascertained at or around admission, the study does not assess SARS-CoV-2-related effect modification, temporality, prediction, or causality.

## 2. Materials and Methods

### 2.1. Study Design and Setting

This was a retrospective secondary analysis of a previously described single-centre cohort, with a cross-sectional primary analysis of the association between documented AF and HF. The study was conducted at the “Victor Babeș” Clinical Hospital of Infectious Diseases and Pneumophthisiology, Timișoara, a tertiary referral centre serving Western Romania. The study period extended from 1 September 2022 to 31 December 2024. The parent cohort and its primary pulmonary embolism analysis have been reported previously [27]; the present manuscript addresses a distinct research question. Reporting followed the STROBE statement for observational studies [29].

### 2.2. Participants

Consecutive adults (aged ≥ 18 years) admitted with SARS-CoV-2 infection confirmed by real-time reverse-transcription polymerase chain reaction on nasopharyngeal swab were eligible. Patients were excluded if the admission was for a non-COVID indication with incidental positivity, if the hospital stay was shorter than 24 h, or if admission records lacked sufficient documentation to ascertain cardiac comorbidity status. A total of 395 patients met all criteria. No sampling or matching was applied.

### 2.3. Variable Definitions

Atrial fibrillation was coded when the admission cardiovascular record contained an explicit AF diagnosis supported by electrocardiographic documentation available to the treating team. The analytic dataset did not retain the date of the qualifying tracing or a uniform pre-admission look-back interval. AF therefore represents AF documented by the admission assessment and may include both previously known and first-detected AF. Paroxysmal, persistent, and permanent labels were inconsistently time-stamped and were not analysed separately.

Heart failure was ascertained from the cardiovascular comorbidity free-text field using a prespecified two-step procedure. Entries were normalized for capitalization, spacing, and Romanian diacritics and searched for explicit diagnostic expressions, including “IC NYHA”, “IC congestivă/IC congestiva”, and “insuficiență cardiacă/insuficienta cardiaca”. The rule-based extraction was implemented during data curation, after which a physician investigator from the study team manually reviewed the original free-text field for all 395 participants. Ambiguous or non-specific entries were classified as HF only when an explicit HF diagnostic term was present; isolated valvular disease, cardiomegaly, ischaemic heart disease, ventricular hypertrophy, or oedema was not sufficient. The manual audit identified one additional explicit HF record missed by the original literal parser, yielding 106 patients with documented HF. A formal blinded dual-reader adjudication and inter-rater agreement assessment were not available. A restrictive sensitivity definition required an explicit NYHA-coded HF entry and identified 102 patients.

Recorded NYHA class was extracted only when a class I–IV designation appeared in the source field. The dataset did not establish whether this class represented stable pre-admission functional status or assessment during acute COVID-19. NYHA results are therefore presented as recorded documentation rather than as a validated measure of chronic HF severity.

The primary adjustment set was specified on clinical and causal grounds before refitting the model. It included age, sex, body mass index, arterial hypertension, ischaemic heart disease, pre-existing type 2 diabetes mellitus, chronic kidney disease, chronic obstructive pulmonary disease, current or former smoking, and prior ischaemic stroke. These variables represent demographic, cardiometabolic, renal, pulmonary, and vascular factors that may be related to both AF and HF. Hypertension required an explicit hypertension-grade entry; five coronary-syndrome entries misplaced in the hypertension field were not coded as hypertension. Chronic kidney disease required an explicit chronic kidney disease designation. Left ventricular hypertrophy was excluded from the primary model because it may lie on the pathway from long-standing hypertension to HF and was added only in sensitivity analysis. Admission oxygen saturation and laboratory variables were used descriptively and were not included in the cross-sectional primary model.

### 2.4. Statistical Analysis

Continuous variables are presented as medians with interquartile ranges and were compared using the Mann–Whitney U test. Categorical variables are presented as counts with percentages and were compared using Fisher’s exact test or the chi-squared test, as appropriate. These univariable comparisons are descriptive.

Because documented HF was common, the primary analysis used a modified Poisson generalized linear model with a log link and robust sandwich variance to estimate adjusted prevalence ratios. AF was the exposure of interest and documented HF was the dependent variable. The model included the prespecified covariates described above. The resulting estimates quantify conditional co-prevalence and are not temporal or causal effects.

Sensitivity analyses comprised logistic regression using the same covariate set, the restrictive NYHA-coded HF definition, addition of left ventricular hypertrophy, an expanded diabetes definition including newly documented diabetes, and exclusion of observations exceeding the conventional Cook’s distance threshold of 4/n.

Model diagnostics included variance inflation factors, centred quadratic terms for age and body mass index, deviance and Pearson goodness-of-fit ratios, standardized residuals, leverage, and Cook’s distance. Logistic-model calibration was evaluated descriptively with the Hosmer–Lemeshow statistic and Brier score. Each of the 12 variables included in the primary model was available for all 395 participants (0 missing for every variable; complete-case *n* = 395), and no imputation was performed. Variable-level missingness for all primary-model variables, sensitivity-analysis variables, and descriptive variables reported in Table 1 is provided in Supplementary Table S1; model diagnostics and sensitivity analyses are reported in Supplementary Table S2.

In-hospital mortality was retained only as an exploratory descriptive outcome. Exact two-sided 95% binomial confidence intervals were calculated for each AF/HF stratum, without between-stratum hypothesis testing. Cardiorespiratory arrest was removed from the revised analysis because the available analytic dataset did not retain sufficient event-level information to adjudicate its definition, temporal sequence, or overlap with death. Analyses were performed in Python 3.12 using SciPy and statsmodels. Two-sided *p* values below 0.05 were considered statistically significant for the primary association; secondary analyses were not adjusted for multiplicity.

### 2.5. Ethics

The study was conducted in accordance with the Declaration of Helsinki and was approved by the Ethics Committee of the “Victor Babeș” Clinical Hospital of Infectious Diseases and Pneumophthisiology, Timișoara (approval no. 11901/16 December 2025). The approval dated 16 December 2025 specifically covered this retrospective secondary analysis and the scientific publication of de-identified data from admissions completed between 1 September 2022 and 31 December 2024. All participants had provided written informed consent at admission for participation and for the use of their de-identified clinical data in research and scientific publications.

## 3. Results

### 3.1. Cohort Characteristics

The cohort comprised 395 patients with a median age of 72.0 years; 213 (53.9%) were male and 208 (52.7%) resided in urban areas. AF was documented in 68 patients (17.2%) and HF in 106 (26.8%). The two conditions coexisted in 26 patients, representing 6.6% of the cohort and 38.2% of patients with AF; documented HF was present in 24.5% of patients without AF. SARS-CoV-2 vaccination had been received by 262 patients (66.3%), with no difference between the AF and non-AF groups (67.6% vs. 66.1%; *p* = 0.888).

Baseline characteristics stratified by AF status are presented in Table 1. Patients with AF were older than those without AF (median 75.8 years, IQR 68.6–83.0, vs. 72.0 years, IQR 65.0–78.4; *p* = 0.018). Documented HF was more frequent in the AF group (38.2% vs. 24.5%; *p* = 0.024). Other baseline differences were modest and should be interpreted descriptively.

Among admission variables, peripheral oxygen saturation was higher in the AF group (median 91.8% vs. 90.6%; *p* = 0.001), whereas fibrinogen was lower (median 3.45 vs. 3.73 g/L; *p* = 0.012). Other measured inflammatory and laboratory variables did not differ significantly. These findings do not establish lower overall COVID-19 severity in patients with AF.

### 3.2. Atrial Fibrillation and Documented Heart Failure

Documented HF was present in 26 of 68 patients with AF (38.2%) and 80 of 327 patients without AF (24.5%). All primary-model variables were complete, so the modified Poisson analysis included all 395 participants and 106 HF events. The unadjusted prevalence ratio was 1.56 (95% CI 1.09–2.23; *p* = 0.014). In the adjusted model, AF remained associated with documented HF (aPR 1.51, 95% CI 1.06–2.16; *p* = 0.022), and age was also associated with HF (aPR 1.02 per year, 95% CI 1.01–1.04; *p* = 0.010).

Sex, body mass index, hypertension, ischaemic heart disease, pre-existing type 2 diabetes, chronic kidney disease, chronic obstructive pulmonary disease, smoking, and prior ischaemic stroke were not statistically significant in the primary model. These null estimates should not be interpreted as evidence that the corresponding conditions are clinically unimportant.

Results were stable across sensitivity analyses. The restrictive NYHA-coded HF definition yielded an aPR of 1.54 (95% CI 1.07–2.23; *p* = 0.021). Logistic regression yielded an adjusted odds ratio of 1.89 (95% CI 1.06–3.37; *p* = 0.031). Adding left ventricular hypertrophy, broadening the diabetes definition, or excluding six observations with Cook’s distance above 4/n did not materially change the AF estimate (Supplementary Table S2).

**Figure 1:**
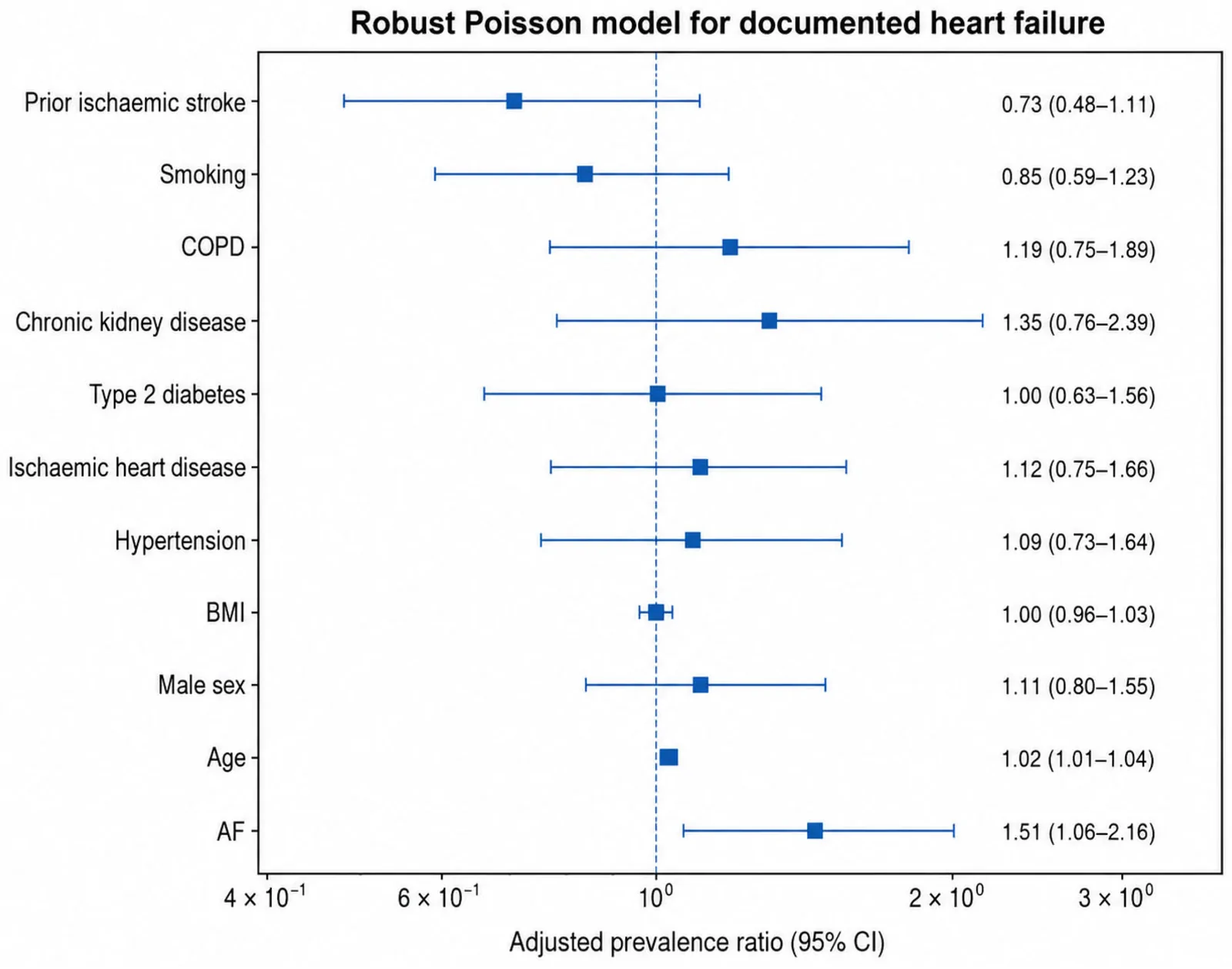
Forest plot of the primary modified Poisson model with robust variance for documented heart failure (*n* = 395; 106 events). Squares denote adjusted prevalence ratios and horizontal lines denote 95% confidence intervals. The x-axis is logarithmic.

The primary model is presented in Table 2 and Figure 1. The maximum variance inflation factor was 2.22. Centred quadratic terms did not indicate non-linearity for age (*p* = 0.841) or body mass index (*p* = 0.139). The deviance/df and Pearson chi-squared/df ratios were 0.69 and 0.75, respectively; no standardized residual exceeded an absolute value of 3. These diagnostics did not identify major instability, although they do not eliminate residual confounding or outcome misclassification.

### 3.3. Recorded NYHA Class

**Figure 2:**
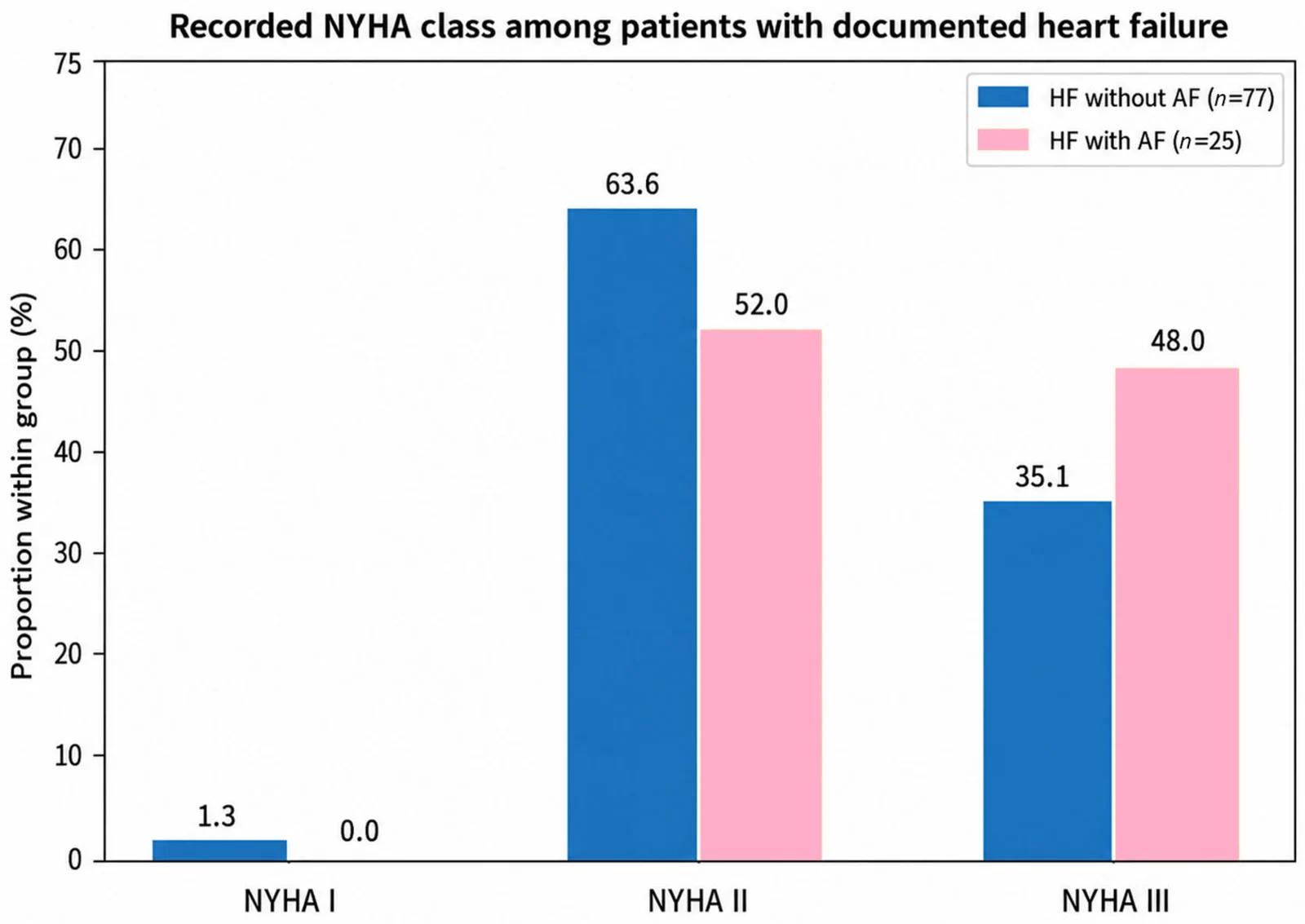
Distribution of recorded New York Heart Association (NYHA) class among patients with documented heart failure, stratified by atrial fibrillation (AF). NYHA class was available in 77 of 80 HF patients without AF and 25 of 26 HF patients with AF; these available-case denominators are used in the figure and percentage calculations. The figure represents recorded documentation and should not be interpreted as a validated measure of chronic HF severity.

Among the 106 patients with documented HF, an NYHA class was recorded for 102 (77 of 80 patients without AF and 25 of 26 patients with AF). Among patients without AF and with an available class, 1 (1.3%) was recorded as NYHA I, 49 (63.6%) as NYHA II, and 27 (35.1%) as NYHA III. Among patients with AF, none was recorded as NYHA I, 13 (52.0%) as NYHA II, and 12 (48.0%) as NYHA III. These values describe charted NYHA documentation only; the dataset could not establish whether the class represented stable pre-admission function or limitation during acute respiratory illness. The distribution is shown in Figure 2.

### 3.4. Exploratory In-Hospital Mortality

Overall in-hospital mortality was 29 of 395 (7.3%). Exact stratum-specific estimates were 5.3% (13/247; 95% CI 2.8–8.8) in patients with neither condition, 9.5% (4/42; 95% CI 2.7–22.6) with AF alone, 10.0% (8/80; 95% CI 4.4–18.8) with HF alone, and 15.4% (4/26; 95% CI 4.4–34.9) with both conditions. The wide and overlapping confidence intervals demonstrate substantial imprecision. These results are provided only as exploratory descriptive data in Supplementary Table S3.

No between-stratum hypothesis test is emphasized, and the data do not support equivalence, risk prediction, or an adjusted prognostic conclusion. Cardiorespiratory arrest was removed from the revised analysis because its event definition, temporal sequence, and overlap with mortality could not be reliably reconstructed from the analytic dataset.

## 4. Discussion

In this cross-sectional secondary analysis of 395 adults hospitalized with COVID-19, AF was associated with a 51% higher adjusted prevalence of documented HF. The principal contribution is a later-pandemic, Eastern European estimate of AF–HF coexistence. There is no evidence that SARS-CoV-2 created or amplified the association.

### 4.1. Interpretation and Clinical Context

The aPR of 1.51 is compatible with the established AF–HF interdependence [10,11,12]. In this cross-sectional dataset, AF should be interpreted as a marker of accumulated cardiovascular substrate and multimorbidity rather than as an isolated causal exposure. Age remained associated with HF, while the other coefficients were imprecise.

This interpretation also aligns with a broader cardiovascular–kidney–metabolic framework, in which AF may represent one component of accumulated multisystem burden rather than an isolated rhythm disorder.

Null estimates for individual comorbidities should not be read as absence of clinical relevance. High prevalence, limited subgroup size, measurement error, and overlap among causal pathways reduce the precision and separability of individual coefficients. Left ventricular hypertrophy was retained as a sensitivity covariate because it may partly mediate the hypertension–HF pathway.

### 4.2. Later-Pandemic Context and Exploratory Outcomes

AF prevalence was 17.2%, higher than pooled estimates from predominantly early-wave cohorts [7,8], plausibly reflecting the advanced age and cardiovascular burden of this tertiary-centre population. Without a non-COVID comparator, neither the prevalence nor the observed association can be attributed specifically to SARS-CoV-2.

Long-term studies have documented post-acute cardiovascular morbidity after COVID-19 [30,31], including regional evidence of persistent cardiopulmonary abnormalities [32]. Although the present study did not assess post-discharge outcomes, future investigations should distinguish pre-existing from new-onset AF [33] (phenotypes that may differ substantially in pathophysiology, prognosis, and therapeutic implications) and determine whether AF represents a risk marker rather than an independent prognostic factor [34].

Frailty and functional reserve are additional unmeasured dimensions. Later-pandemic and large-cohort studies indicate that these dimensions carry prognostic information beyond conventional cardiovascular diagnoses. In a large Italian cohort of patients hospitalized with COVID-19 pneumonia, sex, age, and chronic comorbidity were examined in relation to respiratory impairment and in-hospital outcomes, providing complementary data on cardiovascular risk and prognosis [35]. Using the Clinical Frailty Scale across different pandemic phases, the same group reported that frailty was significantly associated with in-hospital mortality among vaccinated patients in the fourth wave, whereas it did not stratify mortality risk among unvaccinated patients in the first wave [36]. Neither frailty nor functional reserve was available for adjustment in the present analysis.

Consistent with this framework, Askarinejad et al. reported that greater cardiovascular–kidney–metabolic burden was associated with worse outcomes across two prospective AF registries [37]. Our cohort did not formally stage this syndrome, but the adjustment set captured several components of that broader phenotype.

Mortality estimates were imprecise: only four deaths occurred in the AF plus HF stratum, whose exact 95% confidence interval was 4.4–34.9%. These exploratory results are confined to the Supplementary Materials and do not support equivalence, prognosis, or an adjusted outcome claim. The absence of statistically significant differences in mortality between strata should therefore not be interpreted as evidence of an equivalent prognosis; it reflects the small number of events and the consequently limited statistical power of these comparisons.

Larger multicentre studies with sufficient events, prespecified prognostic models, and adjustment for frailty and treatment are required to evaluate clinical outcomes.

### 4.3. Ascertainment and Study Limitations

Disease ascertainment is the principal limitation. AF combined previously known and first-detected cases because the qualifying ECG date was unavailable, and paroxysmal, persistent, and permanent subtypes were not consistently classifiable. HF was derived from audited free text rather than standardized adjudication with systematic echocardiography or natriuretic peptides. The audit improved reproducibility, but no blinded dual-reader validation or inter-rater agreement assessment was available.

Recorded NYHA class also requires caution because the dataset did not establish whether it referred to stable pre-admission function or symptoms during acute COVID-19; dyspnoea and functional limitation may reflect both chronic cardiac disease and acute respiratory illness.

The cross-sectional design precludes temporality and causality. There was no non-COVID comparator, longitudinal rhythm or HF assessment, or systematic classification by left ventricular ejection fraction; cardiac biomarkers and echocardiographic variables were not available for the full cohort.

Structured data on oral anticoagulation, rate or rhythm control, frailty, goals of care, and treatment intensity were unavailable. Residual confounding, single-centre referral patterns, and the later-pandemic setting limit generalizability.

The absence of structured information on oral anticoagulation is a particularly relevant limitation. Anticoagulant exposure may influence thromboembolic and haemorrhagic in-hospital outcomes and represents an unmeasured treatment-related factor relevant to the interpretation of exploratory clinical outcomes. Accordingly, treatment-related residual confounding cannot be excluded, particularly for the exploratory mortality estimates.

### 4.4. Implications and Future Research

Clinically, AF coexisting with documented HF should prompt accurate medication reconciliation and individualized cardiovascular assessment, but the present study does not support a new triage or treatment algorithm. In practice, patients presenting with both AF and documented HF may reasonably warrant closer in-hospital rhythm and haemodynamic monitoring and a more comprehensive cardiovascular evaluation during admission, although the present cross-sectional data are not sufficient to justify a specific change in management. The association is better understood as a signal of cumulative cardiovascular–kidney–metabolic complexity.

Future studies should use time-stamped ECG or telemetry to distinguish pre-existing, admission-detected, and incident AF; standardized HF adjudication with echocardiography and natriuretic peptides; structured frailty assessment; and detailed anticoagulant and rate- or rhythm-control data. A contemporaneous non-COVID comparator would be required to test whether acute SARS-CoV-2 infection modifies the AF–HF relationship.

## 5. Conclusions

In this later-pandemic Romanian cohort of 395 adults hospitalized with COVID-19, AF was associated with a higher prevalence of documented HF after adjustment for demographic, cardiovascular, renal, metabolic, pulmonary, and vascular factors (aPR 1.51, 95% CI 1.06–2.16). The association was robust to alternative HF definitions and model specifications. These findings describe AF–HF coexistence within the cohort and should not be interpreted as evidence that COVID-19 modifies the established relationship, or as proof of temporality, causality, or prognosis.

## Figures and Tables

**Table 1:** Baseline characteristics, admission laboratory values, and in-hospital outcomes stratified by atrial fibrillation status (*n* = 395).

Characteristic	AF (*n* = 68)	No AF (*n* = 327)	*p*
Demographic and anthropometric			
Age, years, median (IQR)	75.8 (68.6–83.0)	72.0 (65.0–78.4)	0.018
Male sex, *n* (%)	36 (52.9)	177 (54.1)	0.894
Body mass index, kg/m^2^, median (IQR)	28.7 (25.5–32.5)	27.9 (23.7–32.2)	0.357
Current or former smoking, *n* (%)	25 (36.8)	109 (33.3)	0.577
Vaccinated against SARS-CoV-2, *n* (%)	46 (67.6)	216 (66.1)	0.888
Comorbidities			
Arterial hypertension, *n* (%)	50 (73.5)	257 (78.6)	0.423
Heart failure, *n* (%)	26 (38.2)	80 (24.5)	0.024
Ischaemic heart disease, *n* (%)	10 (14.7)	65 (19.9)	0.397
Left ventricular hypertrophy, *n* (%)	12 (17.6)	43 (13.1)	0.338
Ischaemic ECG changes, *n* (%)	15 (22.1)	53 (16.2)	0.288
Prior ischaemic stroke, *n* (%)	21 (30.9)	73 (22.3)	0.158
Pre-existing type 2 diabetes mellitus, *n* (%)	12 (17.6)	33 (10.1)	0.092
Chronic kidney disease, *n* (%)	5 (7.4)	13 (4.0)	0.212
Chronic obstructive pulmonary disease, *n* (%)	6 (8.8)	46 (14.1)	0.324
Laboratory values at admission			
Peripheral oxygen saturation, %	91.8 (90.0–94.1)	90.6 (89.0–92.5)	0.001
Fibrinogen, g/L	3.45 (2.84–4.02)	3.73 (3.12–4.26)	0.012
C-reactive protein, mg/L	113.3 (64.9–150.0)	109.5 (47.3–140.5)	0.352
Interleukin-6, pg/mL	14.1 (5.2–28.2)	10.7 (4.2–33.8)	0.549
D-dimer, µg/mL	0.85 (0.47–1.16)	0.71 (0.52–1.10)	0.260
Ferritin, ng/mL	651.6 (361.8–1208.1)	549.0 (241.3–980.1)	0.079
Leukocytes, ×10^9^/L	11.7 (9.2–15.6)	11.8 (7.8–15.2)	0.556
Platelets, ×10^9^/L	259.5 (211.8–305.5)	274.0 (208.0–337.5)	0.413
Serum creatinine, mg/dL	0.89 (0.55–1.31)	0.91 (0.63–1.42)	0.566

AF, atrial fibrillation; ECG, electrocardiogram; IQR, interquartile range. Continuous variables were compared using the Mann–Whitney U test and categorical variables using Fisher’s exact test or the chi-square test, as appropriate.

**Table 2:** Primary modified Poisson model with robust variance for documented heart failure (complete-case *n* = 395; 106 events; 0 missing values for each model variable).

Variable	Adjusted PR	95% CI	*p*
Atrial fibrillation	1.51	1.06–2.16	0.022
Age (per year)	1.02	1.01–1.04	0.010
Male sex	1.11	0.80–1.55	0.526
Body mass index (per kg/m^2^)	1.00	0.96–1.03	0.832
Arterial hypertension	1.09	0.73–1.64	0.678
Ischaemic heart disease	1.12	0.75–1.66	0.583
Pre-existing type 2 diabetes mellitus	1.00	0.63–1.56	0.984
Chronic kidney disease	1.35	0.76–2.39	0.306
Chronic obstructive pulmonary disease	1.19	0.75–1.89	0.466
Current or former smoking	0.85	0.59–1.23	0.386
Prior ischaemic stroke	0.73	0.48–1.11	0.142

## Data Availability

De-identified clinical and laboratory data supporting the findings of this study are available from the corresponding author upon reasonable request, subject to institutional data-sharing policies.

## Supplementary Materials

The following supporting information can be downloaded at: https://www.mdpi.com/article/10.3390/jcm15166470/s1, Supplementary Table S1: Operational definitions and variable-level missingness; Supplementary Table S2: Model diagnostics, sensitivity analyses, and influence checks; Supplementary Table S3: Exploratory in-hospital mortality by atrial fibrillation and heart failure status.

## Abbreviations

The following abbreviations are used in this manuscript:

AF	Atrial fibrillation
aOR	Adjusted odds ratio
BMI	Body mass index
CI	Confidence interval
COPD	Chronic obstructive pulmonary disease
COVID-19	Coronavirus disease 2019
CRP	C-reactive protein
ECG	Electrocardiogram
HF	Heart failure
IL-6	Interleukin-6
IQR	Interquartile range
NT-proBNP	N-terminal pro-B-type natriuretic peptide
NYHA	New York Heart Association
OR	Odds ratio
RT-PCR	Real-time reverse-transcription polymerase chain reaction
SARS-CoV-2	Severe acute respiratory syndrome coronavirus 2
SpO_2_	Peripheral oxygen saturation
STROBE	Strengthening the Reporting of Observational Studies in Epidemiology
aPR	Adjusted prevalence ratio
CKD	Chronic kidney disease
PR	Prevalence ratio
VIF	Variance inflation factor
