# Supplementary material for: Atrial Fibrillation and Documented Heart Failure in Patients Hospitalized with COVID-19: A Secondary Analysis of a Single-Centre Cohort from Western Romania
Source: J Clin Med. 2026 Aug 21;15(16):6470. doi: 10.3390/jcm15166470 (PMC13513572; doi:10.3390/jcm15166470)
Supplement: Supplementary file 1 [file jcm-15-06470-s001.zip › jcm-4483311-supplementary.pdf]

Supplementary Material

Atrial Fibrillation and Documented Heart Failure in Patients Hospitalized with COVID-19: A Secondary Analysis of a Single-Centre Cohort from Western Romania

Supplementary Table S1. Operational definitions and variable-level missingness. All 12 variables included in the primary modified Poisson analysis (AF, documented HF, and the 10 prespecified adjustment covariates) were complete for all 395 participants; no imputation was performed. Variable-level missingness is additionally reported for variables used in sensitivity analyses and for all descriptive variables presented in Table 1.

Variable	Operational definition / ascertainment	Missing
Atrial fibrillation (AF)	Explicit AF diagnosis in the admission cardiovascular record, supported by electrocardiographic documentation available to the treating team. Because the qualifying ECG date and a uniform pre-admission look-back interval were not retained, AF may include both previously known and first-detected AF. Paroxysmal, persistent, and permanent subtypes were not analysed separately because time-stamping was inconsistent.	0
Heart failure (primary outcome definition)	Two-step ascertainment from the cardiovascular free-text field: (1) rule-based search for explicit diagnostic expressions, including "IC NYHA", "IC congestivă/IC congestiva", and "insuficiență cardiacă/insuficienta cardiaca"; and (2) manual physician review of the original free-text field for all 395 participants. Ambiguous or non-specific entries were not coded as HF unless an explicit HF diagnostic term was present. Isolated valvular disease, cardiomegaly, ischaemic heart disease, ventricular hypertrophy, or oedema was insufficient. The manual audit identified one additional explicit HF record missed by the literal parser, yielding 106 patients with documented HF.	0
Heart failure (restrictive sensitivity definition)	Required an explicit NYHA-coded HF entry in the source field; 102 participants met this restrictive definition.	0
Age	Age in completed years at hospital admission.	0
Sex	Biological sex as recorded in the admission record (male/female).	0
Body mass index (BMI)	Weight (kg) divided by height squared (m^2), calculated from admission anthropometric measurements.	0
Arterial hypertension	Required an explicit hypertension-grade entry in the source documentation. Five coronary-syndrome entries misplaced in the hypertension field were not coded as hypertension.	0
Ischaemic heart disease	Documented history of ischaemic heart disease/coronary artery disease in the admission record.	0

Variable	Operational definition / ascertainment	Missing
Pre-existing type 2 diabetes mellitus	Type 2 diabetes mellitus documented before the index admission. An expanded definition that also included newly documented diabetes during admission was used only as a sensitivity analysis.	0
Chronic kidney disease	Required an explicit chronic kidney disease designation in the source documentation.	0
Chronic obstructive pulmonary disease	Documented COPD in the admission record.	0
Current or former smoking	Any documented history of current or former tobacco smoking at admission.	0
Prior ischaemic stroke	Documented history of ischaemic stroke before the index admission.	0
Left ventricular hypertrophy (sensitivity only)	Documented left ventricular hypertrophy. Excluded from the primary model because it may lie on the pathway from long-standing hypertension to HF; added only in sensitivity analysis.	0
Recorded NYHA class	Extracted only when an explicit NYHA class I–IV designation appeared in the source field. Available for 102 of 106 patients with documented HF. The dataset did not establish whether the recorded class represented stable pre-admission functional status or assessment during acute COVID-19.	4 of 106 HF patients
SARS-CoV-2 vaccination	Documented receipt of SARS-CoV-2 vaccination before the index admission.	0
Ischaemic ECG changes	Ischaemic electrocardiographic changes documented on the admission ECG/clinical record.	0
Peripheral oxygen saturation	Peripheral oxygen saturation measured at admission.	0
Fibrinogen	Admission plasma fibrinogen concentration.	0
C-reactive protein	Admission serum C-reactive protein concentration.	0
Interleukin-6	Admission serum interleukin-6 concentration.	0
D-dimer	Admission plasma D-dimer concentration.	0
Ferritin	Admission serum ferritin concentration.	0
Leukocytes	Admission peripheral blood leukocyte count.	0
Platelets	Admission peripheral blood platelet count.	0
Serum creatinine	Admission serum creatinine concentration.	0

Note: SARS-CoV-2 vaccination, ischaemic ECG changes, admission laboratory variables, and peripheral oxygen saturation were used descriptively and were not included in the cross-sectional primary model. Missingness is reported separately for each variable for transparency. A formal blinded dual-reader adjudication and inter-rater agreement assessment for HF ascertainment were not available.

Supplementary Table S2. Model diagnostics, sensitivity analyses, and influence checks. Primary analysis: modified Poisson generalized linear model with log link and robust sandwich variance (complete-case n = 395; 106 documented HF events).

A. Diagnostics for the primary modified Poisson model

Diagnostic	Result	Interpretation
Maximum variance inflation factor (VIF)	2.22	No important multicollinearity signal identified.
Centred quadratic term for age	p = 0.841	No evidence of non-linearity.
Centred quadratic term for body mass index	p = 0.139	No evidence of non-linearity.
Deviance / degrees of freedom	0.69	No evidence of major lack of fit from this diagnostic.
Pearson chi-squared / degrees of freedom	0.75	No evidence of major lack of fit from this diagnostic.
Standardized residuals with ∣value∣ > 3	None	No extreme standardized residuals identified.
Observations with Cook's distance > 4/n	6 observations	Influence sensitivity analysis performed; exclusion did not materially change the AF estimate.
Overall residual / leverage / influence review	No major instability identified	Residual confounding and outcome misclassification cannot be excluded by model diagnostics.

B. Quantitative sensitivity analyses for the AF–HF association

Analysis	Effect measure	Estimate	95% CI	*p* value
Primary modified Poisson model	aPR	1.51	1.06–2.16	0.022
Restrictive NYHA-coded HF definition	aPR	1.54	1.07–2.23	0.021
Logistic regression using the same covariate set	aOR	1.89	1.06–3.37	0.031

C. Additional robustness checks

Robustness check	Result reported in the analysis
Primary modified Poisson model + left ventricular hypertrophy	The AF estimate was not materially changed.
Expanded diabetes definition including newly documented diabetes	The AF estimate was not materially changed.
Exclusion of the 6 observations with Cook's distance > 4/n	The AF estimate was not materially changed.

Notes: aPR = adjusted prevalence ratio; aOR = adjusted odds ratio; CI = confidence interval. The prespecified primary covariate set comprised age, sex, body mass index, arterial hypertension, ischaemic heart disease, pre-existing type 2 diabetes mellitus, chronic kidney disease, chronic obstructive pulmonary disease, current or former smoking, and prior ischaemic stroke. Secondary and sensitivity analyses were exploratory and were not adjusted for multiplicity.

Supplementary Table S3. Exploratory in-hospital mortality by atrial fibrillation and heart failure status. Overall in-hospital mortality was 29 of 395 (7.3%). Exact two-sided 95% binomial confidence intervals are shown. No between-stratum hypothesis testing was performed. These data are descriptive and exploratory; the wide and overlapping confidence intervals do not support equivalence, risk prediction, or an adjusted prognostic conclusion.

Stratum	Deaths / *n*	Mortality (%)	Exact 95% CI	Interpretation
Neither AF nor HF	13 / 247	5.3	2.8–8.8	Reference stratum
AF alone (no HF)	4 / 42	9.5	2.7–22.6	Imprecise
HF alone (no AF)	8 / 80	10.0	4.4–18.8	Imprecise
Both AF and HF	4 / 26	15.4	4.4–34.9	Highly imprecise
Overall cohort	29 / 395	7.3	5.0–10.4	Overall estimate

AF = atrial fibrillation; HF = heart failure; CI = confidence interval. Only four deaths occurred in the AF + HF stratum; therefore, the absence of statistically significant between-stratum differences must not be interpreted as evidence of equivalent prognosis and is compatible with limited statistical power. Cardiorespiratory arrest was removed from the revised analysis because the analytic dataset did not retain sufficient event-level information to adjudicate its definition, temporal sequence, or overlap with death.

Supplementary abbreviations

AF, atrial fibrillation; aOR, adjusted odds ratio; aPR, adjusted prevalence ratio; BMI, body mass index; CI, confidence interval; CKD, chronic kidney disease; COPD, chronic obstructive pulmonary disease; HF, heart failure; LVH, left ventricular hypertrophy; NYHA, New York Heart Association; PR, prevalence ratio; VIF, variance inflation factor.
